# Polydisperse Microparticle Transport and Deposition to the Terminal Bronchioles in a Heterogeneous Vasculature Tree

**DOI:** 10.1038/s41598-018-34804-x

**Published:** 2018-11-06

**Authors:** Mohammad S. Islam, Suvash C. Saha, Tevfik Gemci, Ian A. Yang, Emilie Sauret, Y. T. Gu

**Affiliations:** 1 School of Mechanical and Mechatronic Engineering, Faculty of Engineering and Information Technology, University of Technology Sydney, Ultimo, NSW 2007 Australia; 2 Validation Engineer Specialist, B. Braun Medical Inc, 2525 McGaw Avenue, Irvine, CA USA; 3 Department of Thoracic Medicine, The Prince Charles Hospital, Metro North Hospital and Health Service, and Faculty of Medicine, The University of Queensland, Brisbane, Australia; 4 School of Chemistry, Physics & Mechanical Engineering, Queensland University of Technology, Brisbane, QLD 4001 Australia

## Abstract

The atmospheric particles from different sources, and the therapeutic particles from various drug delivery devices, exhibit a complex size distribution, and the particles are mostly polydisperse. The limited available *in vitro*, and the wide range of *in silico* models have improved understanding of the relationship between monodisperse particle deposition and therapeutic aerosol transport. However, comprehensive polydisperse transport and deposition (TD) data for the terminal airways is still unavailable. Therefore, to benefit future drug therapeutics, the present numerical model illustrates detailed polydisperse particle TD in the terminal bronchioles for the first time. Euler-Lagrange approach and Rosin-Rammler diameter distribution is used for polydisperse particles. The numerical results show higher deposition efficiency (DE) in the right lung. Specifically, the larger the particle diameter (d_p_ > 5 μm), the higher the DE at the bifurcation area of the upper airways is, whereas for the smaller particle (d_p_ < 5 μm), the DE is higher at the bifurcation wall. The overall deposition pattern shows a different deposition hot spot for different diameter particle. These comprehensive lobe-specific polydisperse particle deposition studies will increase understanding of actual inhalation for particle TD, which could potentially increase the efficiency of pharmaceutical aerosol delivery at the targeted position of the terminal airways.

## Introduction

Airborne particles (dust, fumes, smoke, soot, droplets etc.) from various sources, and therapeutic drug particles from metered-dose inhalers (MDIs), dry-powder inhalers (DPIs) and nebulizers, are composed of complex size distributions. Particulates in the ambient air from natural and man-made sources include thoracic and respirable particles^1^, coarse inhalable particles (PM_10_), fine particles (PM_2.5_)^2^, and ultrafine particles. These polydisperse particles are inhaled through the extrathoracic and tracheobronchial airways and down into the terminal bronchioles^3^. Particulate matter (PM_10_) are mostly deposited in the extrathoracic and tracheobronchial airways due to strong inertial impaction and sedimentation^4^; whereas PM_2.5_ and ultrafine particle particulate matter could deposit at the alveolar airways^5–7^. Particulate matter (PM_2.5_) and ultrafine particles may interact with epithelium cells, vessels and submucosa of the airways^8,9^.

A therapeutic drug aerosol may be absorbed by the epithelium cells; and toxic airborne particles occur in different respiratory diseases by producing inflammation in the lung epithelium^10^. A fine particle could cross the alveolar epithelium wall to the interstitial space and finally, contact the blood stream of the lung capillary^2^. Because airborne particle size distribution is mostly polydisperse, these polydisperse particles are inhaled during breathing. Comprehensive polydisperse particle TD data for the extrathoracic and intrathoracic airways is still not available; therefore inclusive polydisperse particle TD for the entire lung model is important to better understand actual particle TD in the lung airways.

Extensive *in vivo* and *in silico* studies have been performed on monodisperse particle TD for both the extrathoracic and intrathoracic regions of the lung^11–19^. The monodisperse particle TD study has improved understanding of airway deposition patterns for the upper airways. Relatively few studies have been conducted on polydisperse particle TD in the lung airways. The ambient and occupational settings of aerosol particulate matter are polydisperse^20^, and polydisperse particles have been associated with adverse respiratory health effects^21^. A series of data on monodisperse aerosol particle TD^22^ has been used to predict polydisperse particle TD in the human lung.

Lognormal size distribution is used for the theoretical study of regional and total deposition. Discrete monodisperse fraction base size distribution shows greater error for regional deposition, especially for particle sizes smaller than 2 µm^23^. A series of monodisperse and polydisperse aerosol particle depositions in a packed bed shows higher total deposition for polydisperse particles than monodisperse particles^20^. The total 1- μm aerosol deposition in the packed bed without a charged neutralizer (an instrument which is used to neutralize the charge of the electronically charged aerosol particle) is 51% for polydisperse particles and 44% for monodisperse particles. An opposite deposition percentage is observed for a charged neutralizer system. The theoretical artificial neural network (ANN) prediction of polydisperse aerosol deposition in a human lung shows a more accurate deposition pattern (<0.025% error) for all regions of the lung^24^. An *In Vivo* study of radioactive polydisperse particle deposition in a child’s respiratory tract shows the extra thoracic deposition pattern^25^; a 72% ± 17% polydisperse aerosol (1 μm-9 μm) deposition was observed in the extrathoracic region.

A recent CFD study by^26^ for a ring less trachea model and a Zygote5 model showed a maximum 68.35% polydisperse particle deposition in the mouth piece and the entire lung model. Particles were injected from the Novolizer dry power inhaler device and spray entered the mouth in a conical fashion. The Zygote5 model considered first seven generations, and a ring structure was used in the trachea. No CFD or experimental study has been conducted which considers the entire branching pattern for polydisperse particle TD for a whole or large-scale lung model. To more accurately assess the polydisperse aerosol TD and respiratory health risk, it is important to investigate the various lobe specific studies.

The present study is the first approach to investigate polydisperse particle TD in the first 17 generations of an asymmetric lung model by considering a likely entire branching pattern. This study calculated the total air flow rate distribution at the five different lobes of a 17-generation model. A detail polydisperse particle TD was performed in the different lobes of the terminal airways and the deposition hot spot at the right and left lung, and different lobes was investigated.

## Numerical Methods

### Governing equations and boundary conditions

The present 17-generation anatomical model is proposed by Schmidt, *et al*.^27^ and is an extension of the CFD study of Gemci, *et al*.^28^ and Islam, *et al*.^6^. Details of the anatomical model can be found in a previous computational study conducted by Islam, *et al*.^6^. An advanced meshing technique was used to generate the unstructured tetrahedral mesh for the complex anatomical model. A fine boundary inflation layer with dense hexahedral mesh was generated near the wall and the final geometry contained approximately 34 million computational cells. Details about the meshing can be found in the Supplementary Section. A complete validation has been performed for the monodisperse particle TD, which can be found in the previous study by Islam, *et al*.^6^. The Large Eddy Simulation (LES) model was used to calculate the air flow in this study. Large eddies are calculated directly; on the contrary, the rest of the smaller eddies are calculated by using a Smagorinsky-Lilly subgrid-scale (SGS) model. The detail of the governing equations and boundary conditions can be found in the Supplementary Sections.

### Particle initial distribution

A parabolic condition^6,29^ is used at the inlet surface. All the particles are injected through the inlet surface; a total 14,800 particles are injected. To check the convergence of the local particle deposition, a different number of particles are injected into the 17-generation model and found the local particle deposition fraction is no more than 1%. In order to minimize the discretization initialization time for the large-scale 17-generation model, a total 14,800 particles are injected for the final simulation. The flow rate is scaled by the face area and particles are injected through the face area. The detail particle properties and governing equations can be found in the Supplementary Section.

**Figure 1:**
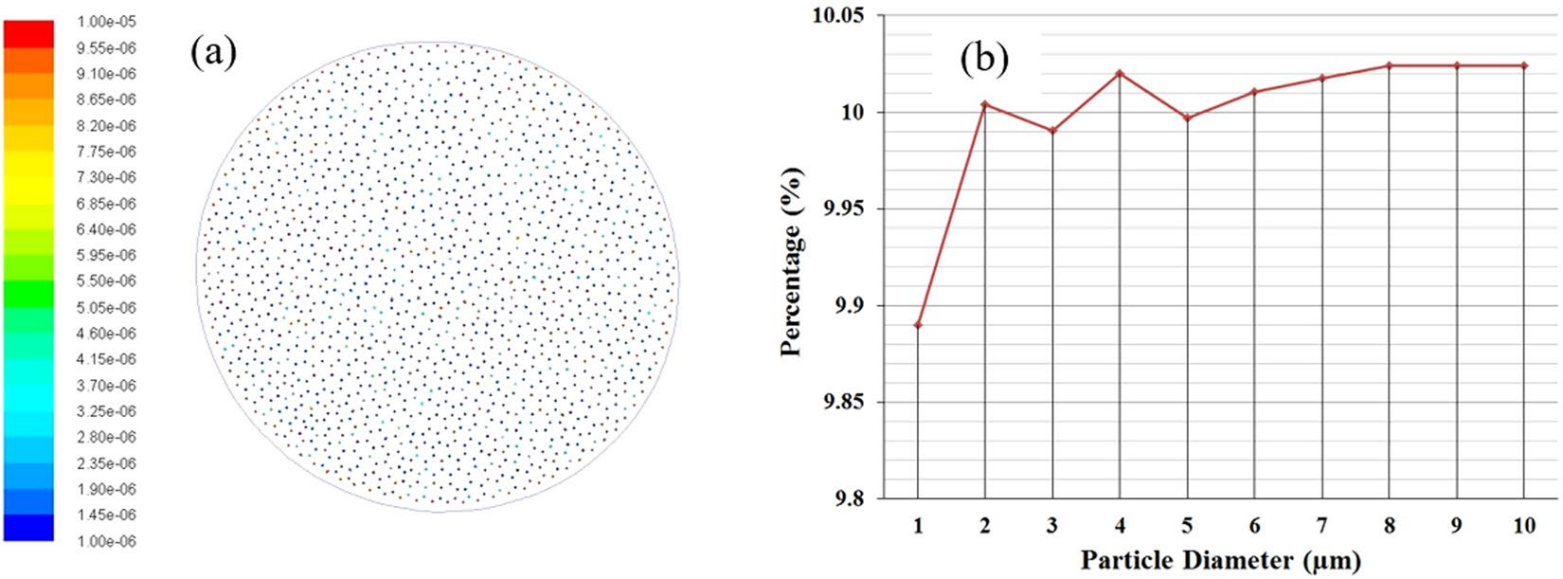
Polydisperse particle distribution, (**a**) schematic diagram of particle initial distribution at inlet and legend shows microparticle (μm) diameter, and (**b**) particle initial distribution curve based on diameter.

Figure 1 shows the polydisperse particle initial distribution at the inlet surface. Figure 1(a) shows the initial position of the different diameter particles; the initial distribution is coloured by a different diameter. The minimum diameter 10^−6^ m, maximum diameters 10^−5^ m, and mean diameter 5×10^−6^ m is used in the Rosin-Rammler distribution method. Figure 1(b) shows the diameter distribution percentage plot. The diameter distribution plot shows a small percentage variation for different diameter particles; however, the initial position of the different diameter particle and the distribution percentage is random.

## Results and Discussion

### Air Flow Simulation

The present study accounts for airflow, polydisperse microparticle TD in a large-scale 17-generation conduit model, and calculates flow rate distribution percentage in the five different lobes. The velocity contour at different selected planes of the upper airways are presented and which can be found in the Supplementary Section.

Table 1 shows the total flow rate distribution at the different lobes of the 17-generation model for different flow rates. The overall flow rate percentage shows that flow distribution is higher in the right lung than in the left. Total flow rate distribution also shows higher flow percentage at the right lower lobe than in all other lobes, and the lower flow distribution at the right middle lobe, compared to other lobes. The highly asymmetric anatomical bifurcating branches significantly influence flow rate distribution at the different lobes. The benchmark experimental data of Cohen, *et al*.^30^ and Horsfield, *et al*.^31^ also show a similar trend of flow rate distribution at the different lobes, which satisfactorily supports the findings of the present study.

**Table 1:** Total flow rate distribution percentage at five different lobes.

	9 lpm	25 lpm	60 lpm	Cohen, *et al*.^30^ 60 lpm	Horsfield, *et al*.^31^
Right Upper Lobe	17.74	18.99	19.07	17.8	21.7
Right Middle Lobe	10.37	10.81	11.1	10.0	9.6
Right Lower Lobe	28.25	27.27	25.86	31.2	23.2
Left Upper Lobe	19.38	19.01	20.05	16.0	20.5
Left Lower Lobe	23.83	23.89	23.92	25.0	24.9
Right Lung	56.36	57.07	56.03	59	54.6
Left Lung	43.21	42.90	43.97	41	45.4

### Particle Transport and Deposition

The current study performs a comprehensive polydisperse particle TD by considering different deposition parameters. A wide range of micro-size particles (1 ≤ d μm ≤ 10) are considered for better prediction of polydisperse particle deposition at the terminal airways.

**Figure 2:**
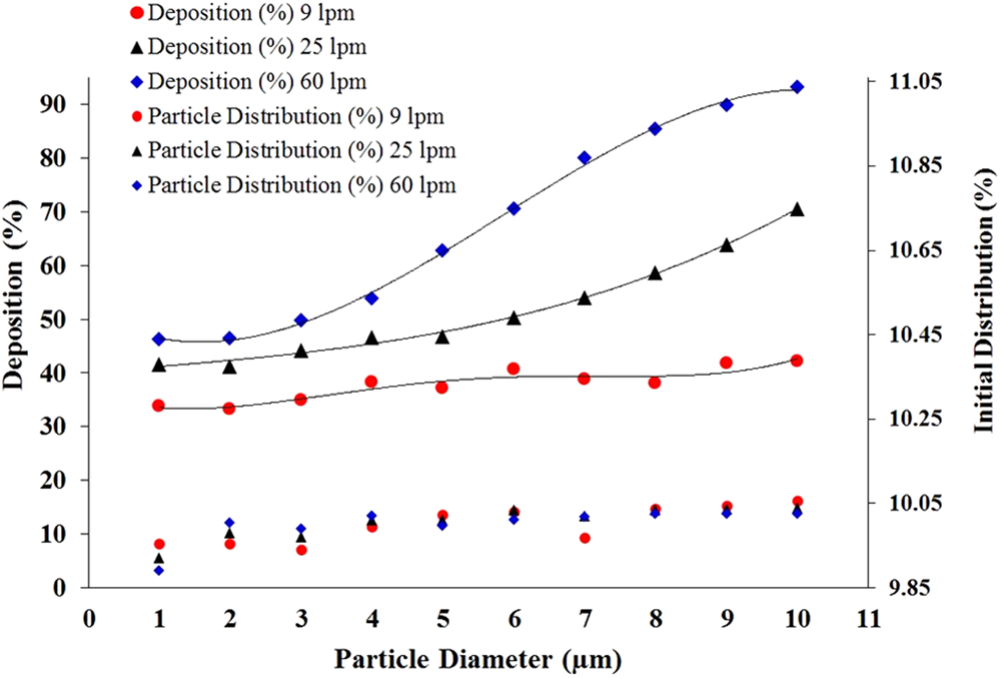
Polydisperse particle initial diameter distribution and total deposition comparison for different flow rates.

Polydisperse particle initial diameter distribution and total deposition comparison for different flow rates is shown in Fig. 2. The primary *y*-axis shows the DE of the deposited particles and the secondary *y*-axis shows the initial diameter distribution of the polydisperse particles. The overall DE of the polydisperse particle for different flow rates shows an increasing trend, except at the 9 lpm flow rate. For the 9 lpm flow rate, the DE shows a fluctuating trend, and about 34% of injected 1- μm particles are deposited, while 42% of injected 10- μm particles are deposited. The DE for 25 lpm and 60 lpm flow rates shows an increasing trend, irrespective of particle diameter. For a 60 lpm flow rate, 46.3% of 1- μm-sized particles are deposited and 93.1% of 10- μm sized particles are deposited. The calculated third order polynomial trend line fits comfortably with the DE curve for different flow rates. The third order analytical DE equation for the 60 lpm flow rate is:

1 $$y=-0.153{x}^{3}+2.6851{x}^{2}-7.3543x+51.291$$

The calculated *R*^2^ value is 0.9985, which clearly supports the curve fitting.

The third order analytical DE equation for the 25 lpm flow rate is;

2 $$y=0.0224{x}^{3}-0.0273{x}^{2}+1.0808x+40.124$$

with *R*^2^ value of 0. 9958, and the 4^th^ order analytical DE equation for 9 lpm flow rate is;

3 $$y=0.0159{x}^{4}-0.3377{x}^{3}+2.325{x}^{2}-4.7352x+36.287$$

with the calculated *R*^2^ value is 0.8753.

The calculated third order polynomial analytical equation can be used for the validation purpose as it fits nicely with the present results. The analytical equations can also be used to predict the overall deposition pattern for a large scale model.

**Figure 3:**
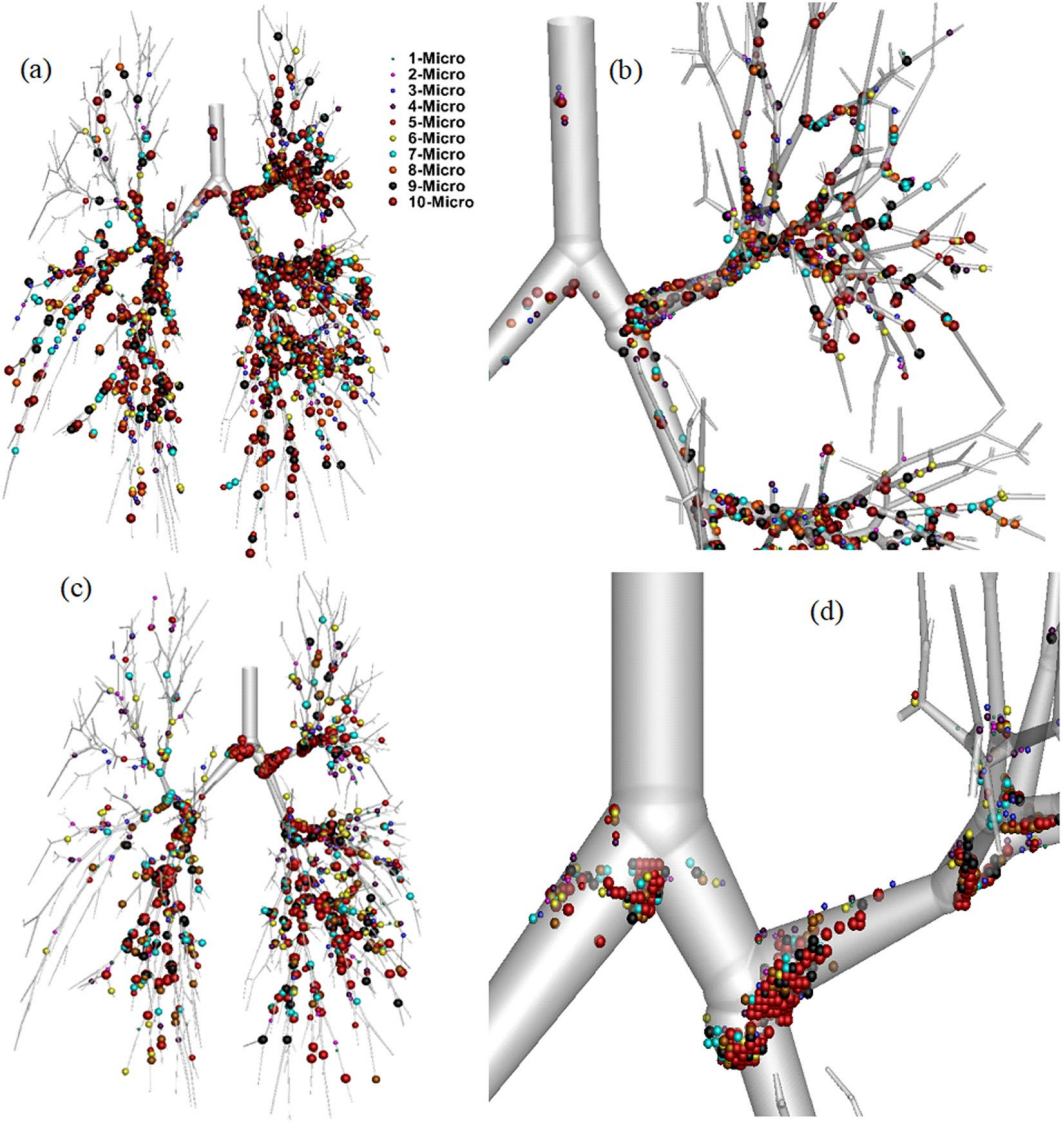
Polydisperse particle deposition pattern in a 17-generation anatomical model for different flow rates: (**a**) overall deposition pattern for 9 lpm flow rate, (**b**) enlarged portion of right upper lobe and right middle lobe, (**c**) overall deposition pattern for 60 lpm flow rate, and (**d**) enlarged portion of first and second bifurcation area.

Figure 3 displays the polydisperse particle deposition pattern for a 17-generation whole lung model by examining the entire possible branching pattern. A wide range of micro-diameter particles (1 μm ≤ dp ≤ 10 μm) is considered in the present study, in which diameter is shown in different sizes and colours. Figure 3(a,c) show polydisperse particle deposition scenario for 9 lpm and 60 lpm flow rates respectively. Figure 3(b) shows enlarged deposition in the right upper lobe and part of the right middle lobe. Figure 3(d) shows the deposition pattern in the bifurcation area of the upper airway. The overall deposition pattern shows higher deposition concentration in the first few generations. At the 9 lpm flow rate, a lesser number of larger diameter particle (d_p_ > 5 μm) are deposited in the bifurcation area of the first and second generation compared to 60 lpm flow rate. Figure 3(b) shows a significant number of particles deposited in the bifurcation wall at the 9 lpm flow rate. For the 60 lpm flow rate, on the contrary, a significant amount of larger diameter (d_p_ > 7 μm) particles are deposited in the bifurcation angle of the first and the second generation than in the 9 lpm flow rate. Figure 3(d) illustrates that fewer particles are deposited in the tracheal wall and the bifurcation wall at the 60 lpm flow rate. The enlarged deposition example also shows that smaller diameter particles are deposited mainly in the bifurcation wall, not at the carinal angle. Figure 3(a,d) show a noticeable amount of smaller diameter particles (d_p_ < 5 μm) deposited at the terminal airways for both flow rates. Moreover, a significant number of smaller diameter particles (d_p_ < 5 μm) are deposited at the terminal airways of the 17-generation model in the 25 lpm flow rate; the deposition figure can be found in the Supplementary Section. The reason for this type of deposition is microparticle inertia, and the inertial impaction is the dominant mechanism. With increased particle diameter, the momentum relaxation time *τ*_*m*_ = *ρ*_*p*_*d*_*p*_^2^*C*_*s*_*/18μ* significantly increases and inertia becomes the vital component for the deposition^32^. Because of the higher inertia, larger diameter particles are deposited at the carinal angle area. A detailed discussion of the mechanism can be found in the authors’ previous work^6^. Inertial parameter (IP) can be defined as;

4 $$IP=\rho {{d}_{p}}^{2}Q$$

where ρ is the density, *d*_*p*_ is the particle diameter and *Q* is the flow rate.

According to eqn. 4, inertial parameter value is increased regardless of flow rate and particle diameter. The inertial parameter value is significantly higher with a higher flow rate and larger diameter particles, which ultimately indicates that inertial impaction is dominant for larger diameter particles and high flow rates. To investigate the polydisperse particle deposition hot spot, deposited particle concentration curve has been calculated. The density curve and the detail discussion can be found in the Supplementary Section.

For better understanding of the polydisperse particle deposition, DE in the right lung and left lung has been investigated and third order analytical polynomial equations are derived from the DE curve. The detail discussion and the developed equations can be found in the Supplementary Section.

**Figure 4:**
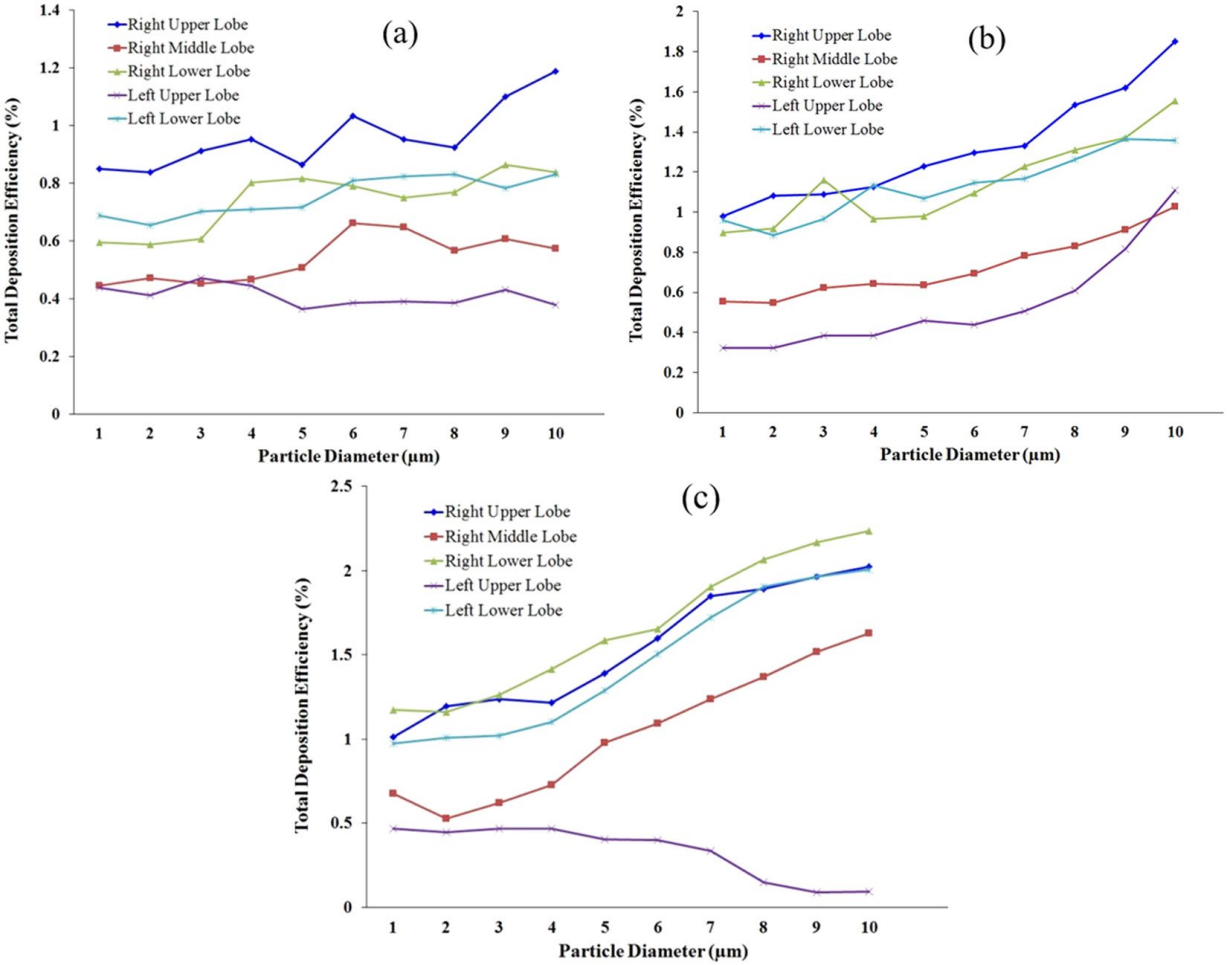
Lobar DE comparison for polydisperse particle: (**a**) 9 lpm flow rate, (**b**) 25 lpm flow rate, (**c**) 60 lpm flow rate.

Figure 4 shows the lobar DE of a polydisperse particle for different flow rates. The DE for various diameter particles in the five different lobes is calculated. Figure 4(a) shows the lobar DE for a 9 lpm flow rate; different colours represent various lobes. The overall deposition DE shows higher deposition at the right upper lobes and lower deposition at the left upper lobes. The overall DE trend at the different lobes is fluctuating, which could reveal the size-specific deposition pattern at different lobes. Figure 4(a) shows that larger diameter particles are deposited mainly at the right upper lobes. Figure 4(a) also shows that the deposition concentration of 4 μm ≤ d_p_ ≤ 6 μm diameter particles are higher at the right lower lobes. However, for a 9 lpm flow rate, particles ranging from 6 μm ≤ d_p_ ≤ 8 μm diameter are deposited mainly at the left lower lobes. The DE curve also illustrates that for lower flow rates, 6 μm and 7 μm diameter particle deposition concentrations are higher at the right middle lobe. Figure 4(b) shows the lobar deposition pattern for different diameter particles in five different lobes at 25 lpm flow rates. The overall deposition pattern shows higher DE at the right upper lobe and lower DE at the left upper lobe. Figure 4(c) shows that the DE at the right lower lobe is higher, and lower at the left upper lobe. Figure 4(c) shows that the DE at the left upper lobe decreases with increased particle diameter; the main reasons for this are gravitational force and the anatomical structure of the left upper lobe. Because of gravity and the higher inertia of the microparticle, fewer micro-diameter particles enter into the left upper lobe.

**Figure 5:**
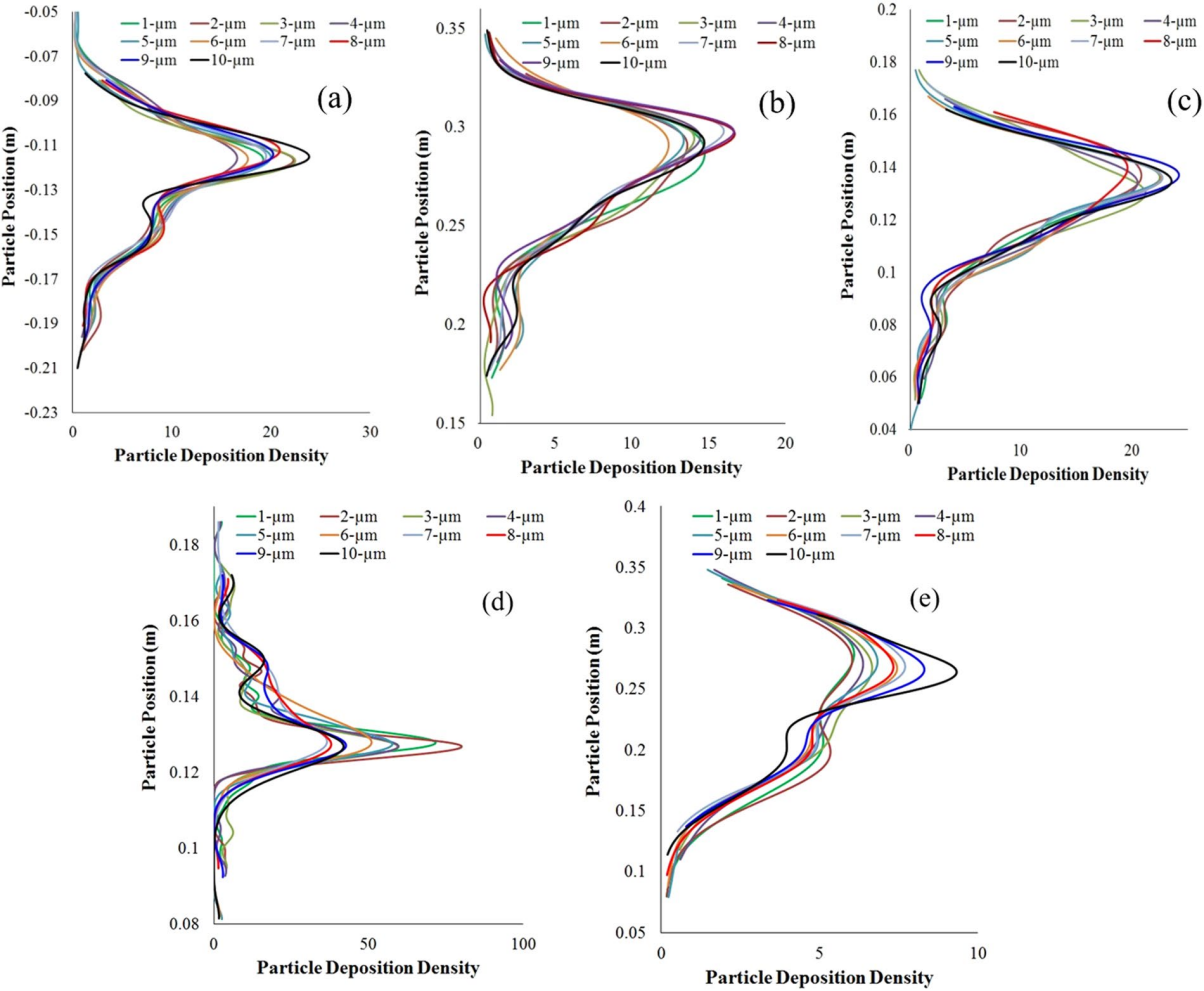
Poly-disperse particle deposition density comparison during 9 lpm flow rate at (**a**) right upper lobe, (**b**) right middle lobe, (**c**) right lower lobe, (**d**) left upper lobe, (**e**) left lower lobe.

The lobar deposition density comparison for different diameter particles reveals different deposition hot spots for various lobes, as shown in Fig. 5. At the right upper lobes, upper generations are the deposition hot spots and 10- μm diameter particle deposition concentration is higher than in particles of different diameters. On the contrary, the deposition density of the 7 μm ≤ d_p_ ≤ 9 μm diameter particle is higher at the upper generation of the right middle lobe. Figure 5(d) shows different deposition hot spots at the left upper lobe for particles of different diameters.

Table 2 shows deposition hot spot for different diameter particle at 9lpm flow rate. The lobe-specific deposition concentration comparison for polydisperse particles significantly increases understanding of the pharmaceutical particle deposition pattern at the terminal airways, which could advance respiratory health risk assessments.

**Table 2:** Deposition hot spot for different diameter particle at 9 lpm flow rate.

Diameter	RUL	RML	RLL	LUL	LLL
1	G3–G5	G4–G6	G4–G5	G4	G4–G6
2	G2–G3	G5	G5, G8	G3–G4	G5
3	G2–G3	G5–G6	G6	G3	G6
4	G3–G4	G4–G6	G5–G7	G3–G4	G4–G5
5	G2–G4	G5	G4–G6	G3	G3–G5
6	G4	G6	G4–G6	G3	G3–G4
7	G3–G4	G4–G5	G4–G5	G3–G4	G3–G4
8	G3–G5	G4–G5	G4–G5	G2–G3	G4
9	G2–G5	G4–G6	G4–G6	G3–G5	G4
10	G2–G5	G4–G6	G4–G6	G2–G3	G3–G5

(The trachea and the first two bifurcating branches are treated as G1 (detail definition figure can be found in Supplementary Section) and follows for the rest of the model. Particles are deposited throughout the 17 generations and the table shows only the hot spots).

## Conclusions

A comprehensive air flow particle TD study has been performed in this study. The present large-scale respiratory model illustrates detail polydisperse particle TD for different flow rates using the Rosin-Rammler diameter distribution technique for polydisperse particle injection. The following conclusions can be drawn from the resulting study:

- Total flow rate distribution at the right lung is 1.3 times higher than that in the left lung and the total flow rate distribution at the lower lobes is higher than in the upper lobes. Specifically, minimum flow rate percentage is at the right middle lobe.
- Larger particle (d_p_ > 5 μm) deposition concentration is higher at the bifurcation area (carinal angle) of the first couple of generations, whereas smaller diameter particle (d_p_ < 5 μm) deposition concentration is higher at the bifurcation wall of the anatomical model.
- DE efficiency of the polydisperse particle is higher in the right lung than in the left lung, regardless of the flow rates.
- Polydisperse particle DE at the right upper lobe is higher compared to other lobes, except at 60 lpm flow rate, where DE is higher at the right lower lobe. Lower DE is observed at the left upper lobe, regardless of the flow rates.
- A different deposition hot spot is observed in the right and left lung for different diameter particles. At a 9lp flow rate, the deposition hot spot is in the upper and middle area of the right lung, and at the middle of the left lung. At 60 lpm flow rate, different deposition hot spots are observed for various diameter particles.
- Different deposition hot spots are observed at the different lobes of the 17-generation anatomical model for different diameter particles and flow rates.
- New analytical equations for the right and left lung DE, for different flow rates, have been developed and can be used to predict the DE for an entire lung model.

The present CFD study is the first comprehensive approach to demonstrate the polydisperse particle TD in different lobes of a large scale vasculature tree. The advanced CFD model performed inclusive lobar deposition and found different deposition hot spot for various deposition parameters. The present model developed new analytical equations for the deposition in the right lung, left lung and different lobes, which would potentially provide overall deposition understanding for a large scale model. The detail flow rate distribution calculation at different lobes will contribute to knowledge of air flow transport for a whole lung airway. Numerical predictions offer proven approximation of therapeutic aerosol particle deposition in the terminal airways of different lobes, generated from nebulizers, dry power inhalers and metered dose inhalers. The complete lobe-specific polydisperse particle TD study, together with clinical observations, will increase knowledge of site-specific drug delivery into the lower airways and the alveolar region of the lung. Detailed numerical prediction for lobar deposition will aid the pharmaceutical industry in the design of more efficient lobe-specific drug delivery and potentially advance the entire drug delivery sector. A more advanced, patient-specific case study will follow.

## Electronic supplementary material

Supplementary Information

## References

[1] Brown JS, Gordon T, Price O, Asgharian B (2013). Thoracic and respirable particle definitions for human health risk assessment. Particle and fibre toxicology.

[2] Chen, J. *et al*. A review of biomass burning: Emissions and impacts on air quality, health and climate in China. *Science of The Total Environment* (2016). 10.1016/j.scitotenv.2016.11.025 27908624

[3] Zhang Z, Kleinstreuer C (2004). Airflow structures and nano-particle deposition in a human upper airway model. Journal of computational physics.

[4] Islam, M. S., Saha, S. C., Sauret, E., Gu, Y. & Ristovski, Z. In Proceedings of the International Conference on Computational Methods. (Scientech Publisher llc, USA.).

[5] Islam, M. S., Saha, S. C., Sauret, E. & Gu, Y. Numerical investigation of diesel exhaust particle transport and deposition in up to 17 generations of the lung airway. *20th Australasian Fluid Mechanics Conference, Perth, Australia* **5**–**8** December (2016).

[6] Islam MS, Saha SC, Sauret E, Gemci T, Gu Y (2017). Pulmonary aerosol transport and deposition analysis in upper 17 generations of the human respiratory tract. Journal of Aerosol Science.

[7] Koullapis, P., Hofemeier, P., Sznitman, J. & Kassinos, S. An efficient computational fluid-particle dynamics method to predict deposition in a simplified approximation of the deep lung. *European Journal of Pharmaceutical Sciences* (2017). 10.1016/j.ejps.2017.09.016 28917963

[8] Gehr, P. & Heyder, J. *Particle-lung interactions*. (CRC Press, 2000).

[9] Islam, M. S. *et al*. Ultrafine Particle Transport and Deposition in a Large Scale 17-Generation Lung Model. *Journal of Biomechanics* (2017). 10.1016/j.jbiomech.2017.08.028 28916396

[10] Laumbach RJ, Kipen HM (2012). Respiratory health effects of air pollution: update on biomass smoke and traffic pollution. Journal of allergy and clinical immunology.

[11] Farkas Á, Balásházy I (2008). Quantification of particle deposition in asymmetrical tracheobronchial model geometry. Computers in Biology and Medicine.

[12] Farkas Á, Balásházy I (2007). Simulation of the effect of local obstructions and blockage on airflow and aerosol deposition in central human airways. Journal of Aerosol Science.

[13] Longest PW, Xi J (2007). Effectiveness of direct Lagrangian tracking models for simulating nanoparticle deposition in the upper airways. Aerosol Science and Technology.

[14] Inthavong K, Tian L, Tu J (2016). Lagrangian particle modelling of spherical nanoparticle dispersion and deposition in confined flows. Journal of Aerosol Science.

[15] Asgharian B, Hofmann W, Bergmann R (2001). Particle deposition in a multiple-path model of the human lung. Aerosol Science & Technology.

[16] Balásházy I, Hofmann W, Martonen TB (1991). Inspiratory particle deposition in airway bifurcation models. Journal of Aerosol Science.

[17] Kim CS, Iglesias AJ (1989). Deposition of inhaled particles in bifurcating airway models: I. Inspiratory deposition. Journal of Aerosol Medicine.

[18] Kim, C. Ultrafine particle deposition in a double bifurcation tube with human G3–G5 airway geometry. (US EPA, Internal Report, 2002).

[19] Lizal, F. *et al*. Experimental methods for flow and aerosol measurements in human airways and their replicas. *European Journal of Pharmaceutical Sciences* (2017). 10.1016/j.ejps.2017.08.021 28842353

[20] Rosati JA, Leith D, Kim CS (2003). Monodisperse and polydisperse aerosol deposition in a packed bed. Aerosol Science & Technology.

[21] Dockery DW (1993). An association between air pollution and mortality in six US cities. New England journal of medicine.

[22] Diu C, Yu C (1983). Respiratory tract deposition of polydisperse aerosols in humans. The American Industrial Hygiene Association Journal.

[23] Ferron G, Karg E, Peter J (1993). Estimation of deposition of polydisperse hygroscopic aerosols in the human respiratory tract. Journal of aerosol science.

[24] Nazir J, Barlow DJ, Jayne Lawrence M, Shrubb I (2005). Artificial neural network prediction of the patterns of deposition of polydisperse aerosols within human lungs. Journal of pharmaceutical sciences.

[25] Albuquerque-Silva I (2014). Particle deposition in a child respiratory tract model: *in vivo* regional deposition of fine and ultrafine aerosols in baboons. PloS one.

[26] Kannan RR (2017). Pharmaceutical aerosols deposition patterns from a Dry Powder Inhaler: Euler Lagrangian prediction and validation. Medical engineering & physics.

[27] Schmidt A (2004). A digital reference model of the human bronchial tree. Computerized Medical Imaging and Graphics.

[28] Gemci T, Ponyavin V, Chen Y, Chen H, Collins R (2008). Computational model of airflow in upper 17 generations of human respiratory tract. Journal of Biomechanics.

[29] White, F. M. Fluid mechanics. 5th. Boston: McGraw-Hill Book Company (2003).

[30] Cohen BS, Sussman RG, Lippmann M (1990). Ultrafine particle deposition in a human tracheobronchial cast. Aerosol Science and Technology.

[31] Horsfield K, Dart G, Olson DE, Filley GF, Cumming G (1971). Models of the human bronchial tree. Journal of applied physiology.

[32] Tsuda, A., Henry, F. S. & Butler, J. P. Particle transport and deposition: basic physics of particle kinetics. *Comprehensive Physiology* (2013). 10.1002/cphy.c100085 PMC4398662 24265235

