# Supplementary material for: Polydisperse Microparticle Transport and Deposition to the Terminal Bronchioles in a Heterogeneous Vasculature Tree
Source: Sci Rep. 2018 Nov 6;8:16387. doi: 10.1038/s41598-018-34804-x (PMC6219544; doi:10.1038/s41598-018-34804-x)
Supplement: Supplementary file 1 — Supplementary Information [file 41598_2018_34804_MOESM1_ESM.docx]

**Polydisperse Microparticle Transport and Deposition to the Terminal Bronchioles in a Heterogeneous Vasculature Tree**

Mohammad S. Islam^1,4^, Suvash C. Saha^1, *^, Tevfik Gemci^2^, Ian A. Yang^3^, Emilie Sauret^4^, Y.T. Gu^4^

^1^*School of Mechanical and Mechatronic Engineering, Faculty of Engineering and Information Technology, University of Technology Sydney, Ultimo NSW 2007, Australia*

^2^*Validation Engineer Specialist, B. Braun Medical Inc., 2525 McGaw Avenue, Irvine, CA,USA*

^3^ *Department of Thoracic Medicine, The Prince Charles Hospital, Metro North Hospital and Health Service, and Faculty of Medicine, The University of Queensland, Brisbane, Australia.*

^4^*School of Chemistry, Physics & Mechanical Engineering, Queensland University of Technology Brisbane QLD 4001, Australia*

**Supplementary Info File**

**Numerical Methods**

**Governing equations and boundary conditions**

| **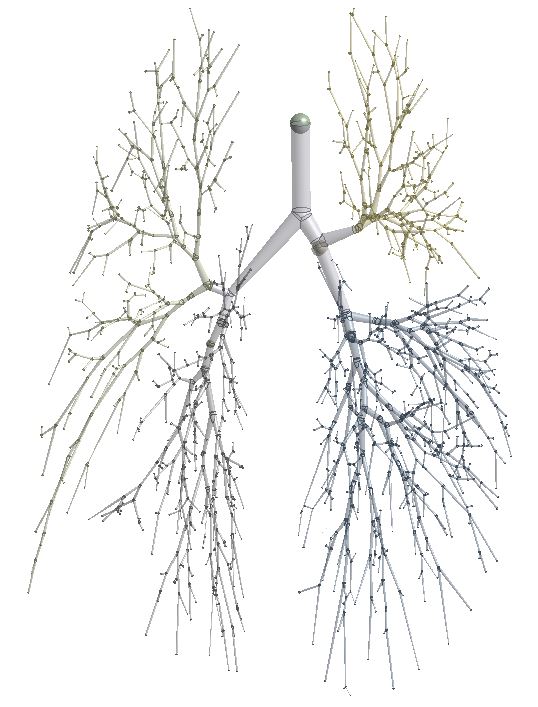**  (a) | **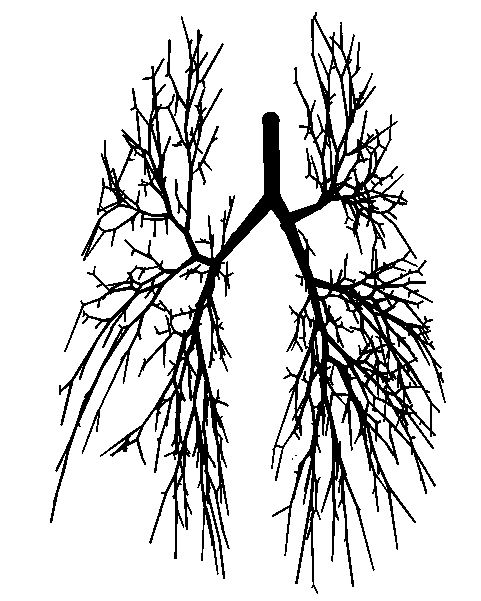**  (b) |
| --- | --- |
| **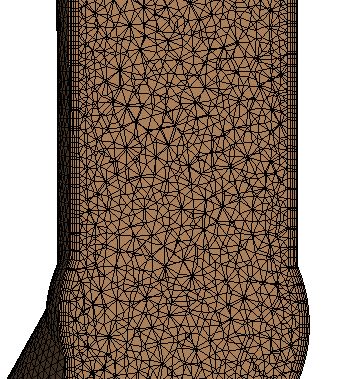**  (c) | **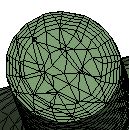**  (d) |

**Supplementary Figure** 1: 17-generation reconstructed model and mesh: (a) reconstructed anatomical model, (b) anterior view of the generated mesh, (c) cross sectional view of the mesh at trachea, (d) mesh at outlet.

The 17-generation conduit model, and the generated mesh, are shown in **Supplementary Figure 1**. The fine boundary inflation layer mesh with hexahedral element is generated at the wall throughout the model. At the bifurcation area, a dense tetrahedral element is used for the complex flow field. A proper grid refinement test (**Supplementary Figure 2**) has been conducted and the final geometry contains approximately 34 million unstructured cells.

**
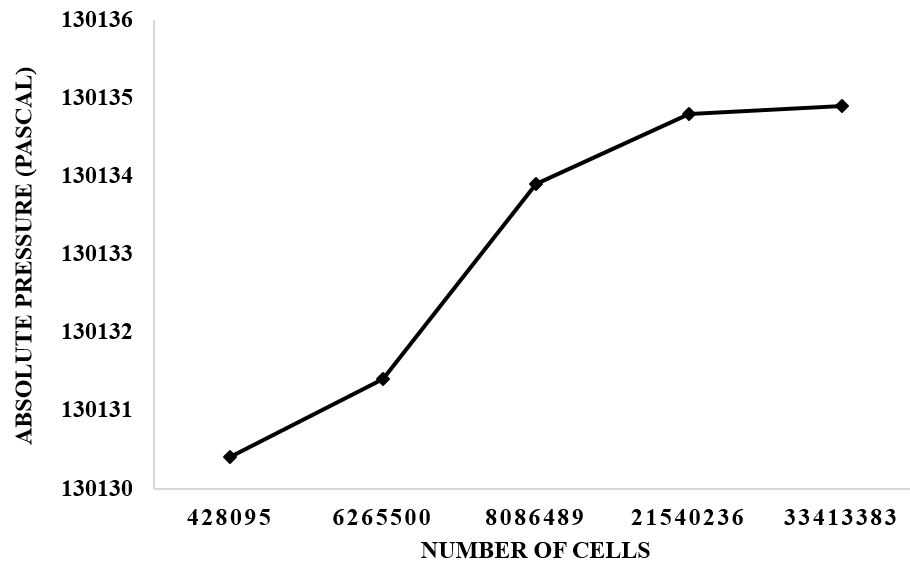
**

**Supplementary Figure** 2: Grid refinement test for the generated unstructured cells.

Mass and the momentum equations are solved to calculate air flow into the computational domain.

$\frac{\partial\rho}{\partial t}+\nabla\cdot\left(\rho\vec{\text{v}}\right)()=S_{m}$ (1)

where ρ is density, $\vec{\text{v}}$ is air velocity vector, and *S_m_* is the mass source term.

(2)

where, $\rho\vec{g}$ is gravitational body force, *p* is the static pressure for air, and $\vec{F}$is external body (particle-fluid interaction) force.

The pressure-velocity coupling scheme, SIMPLE ([Kannan *et al.*, 2017](#_ENREF_20)) is used in the present study. The SIMPLE algorithm is the relation between the velocity and pressure correction to implement mass conservation and obtain the pressure field. The pressure correction ($p'$) equation in the cell can be defined as

(3)

where the source term b is the net flow into the cell.

$b=\sum_{f}^{N_{faces}} J_{f}^{*}A_{f}$ (4)

where $J_{f}^{*}$is the resulting face flux.

The second order pressure and upwind momentum spatial discretization is used in the present study.

For a spherical particulate *i*, drag force is usually the domain force leading the particulates in airways, which can be expressed as:

(5)

where $C_{D}$is the drag coefficient, $d_{p}$is the diameter of the particle, and $\vec{\text{v}}_{\text{p}}$is the particle velocity. The individual particle motion *i* is modelled using Newton’s second law:

(6)

and the particle position is:

$\frac{\partial x_{\text{p}}}{\partial t}=\vec{\text{v}}_{\text{p}}$ (7)

Velocity inlet and pressure outlet boundary conditions are used in the present study. In almost all of the published computational and experimental studies have considered uniform or parabolic ([Balásházy *et al.*, 2003](#_ENREF_2); [Longest and Vinchurkar, 2007](#_ENREF_11); [Sohrabi *et al.*, 2017](#_ENREF_17); [Zhang and Kleinstreuer, 2001](#_ENREF_18); [Zhang *et al.*, 2001](#_ENREF_22)) inlet condition for airflow and particle transport. However, the inlet velocity profile is quite complex for a realistic case depending on the different physical and geometrical conditions. The present study considered a fully developed parabolic inlet condition (White, 2003) for the particle transport and deposition in a large-scale model. Zero pressure is used at the outlet ([Gemci *et al.*, 2008](#_ENREF_13); [Koullapis *et al*., 2016](#_ENREF_24); [Sohrabi *et al*., 2017](#_ENREF_32)) of the present model. A no-slip boundary condition ([Longest and Xi, 2007](#_ENREF_27)) is used at the wall surface and the airway wall is stationary.

**Particle Initial Distribution**

Diesel exhaust particles are considered and the particle density 1,100 kg/m^3^ is used. The spherical drag law is used for the particle. Previously known as the Weibull distribution, the Rosin and Rammler distribution ([Rosin, 1933](#_ENREF_30)) is used to approximate the aerosol particle size distribution.

$f(x;P_{80},m)=\left\{\begin{matrix} 1-e^{\ln(0.2)(\frac{x}{P_{80}})^{m}} x\geq0; \\ 0 x<0, \end{matrix}\right.$ (8)

where, *x* is the particle size, *P_80_* is the 80^th^ percentile of the particle size distribution, *m* is the spreading distribution parameter.

The inverse distribution is:

(9)

Where *F* is the mass fraction.

The Rosin-Rammler distribution in Ansys (17.2) Fluent is the form of the Weibull distribution and requires the initial velocity, total flow rate, maximum, minimum and mean diameter, spread parameters. The spread parameter can be computed from the analytical equations

$n=\frac{\ln(-\ln[Y_{d}])}{\ln\left(\frac{d}{\bar{d}}\right)()}$ (10)

where *Y_d_* is the mass distribution function. The final Rosin-Rammler distribution function used is:

(11)

**Results and Discussion**

**Air Flow Simulation**

| **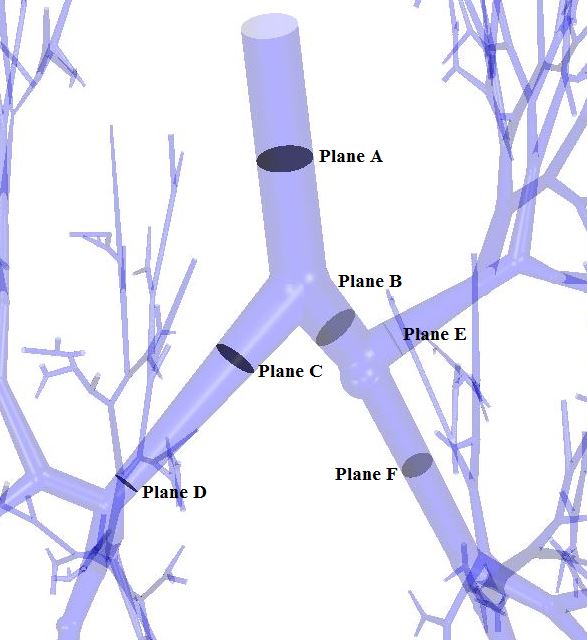**  (E)  (C)  (A) | **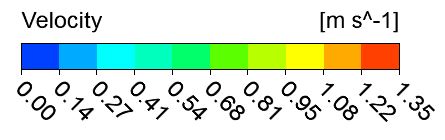** | |
| --- | --- | --- |
|  | **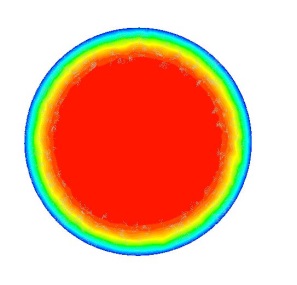** | **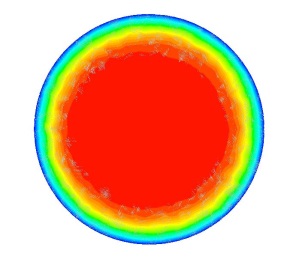**  (B) |
|  | **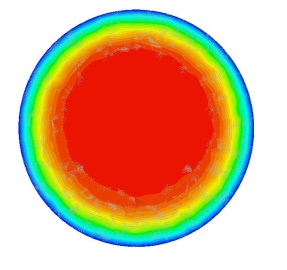**  (D) | **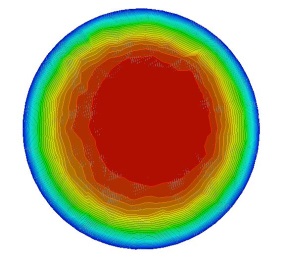**  (F) |
|  | **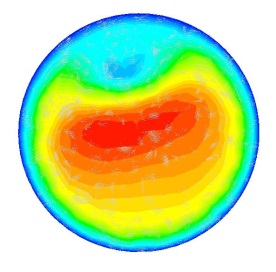** | **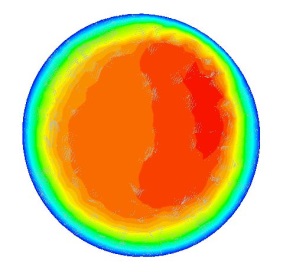** |

**Supplementary Figure** 3: Velocity contour in upper airways of 17-generation model at 9lpm flow rate. Left panel is randomly selected plane; right panel is velocity contours for different planes.

**Supplementary Figure** 3 shows the velocity contours in the first few generations of the 17-generation model at 9 lpm flow rate. The randomly selected plane in the upper airways shows the velocity contour at the right and left lung. The Reynolds number at the trachea is about 730, indicating that laminar flow prevails at this level. The velocity contours at the position of A, B, C, D show the fully developed parabolic flow. The velocity contour at E shows a vortex generated due to the strong change of the cross-sectional area. The complicated asymmetric branching pattern and centrifugally-induced pressure gradient also influence the formation of the velocity contour.

**Particle transport and Deposition**

| 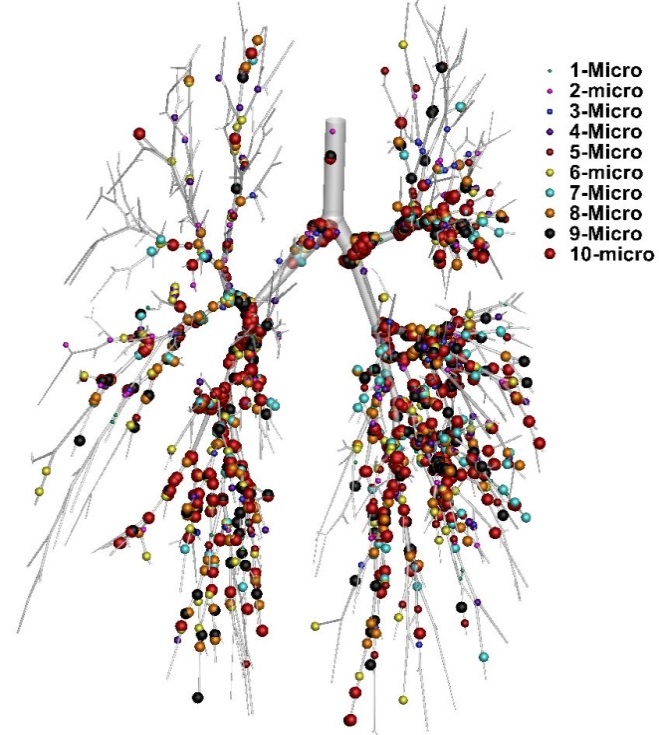 | 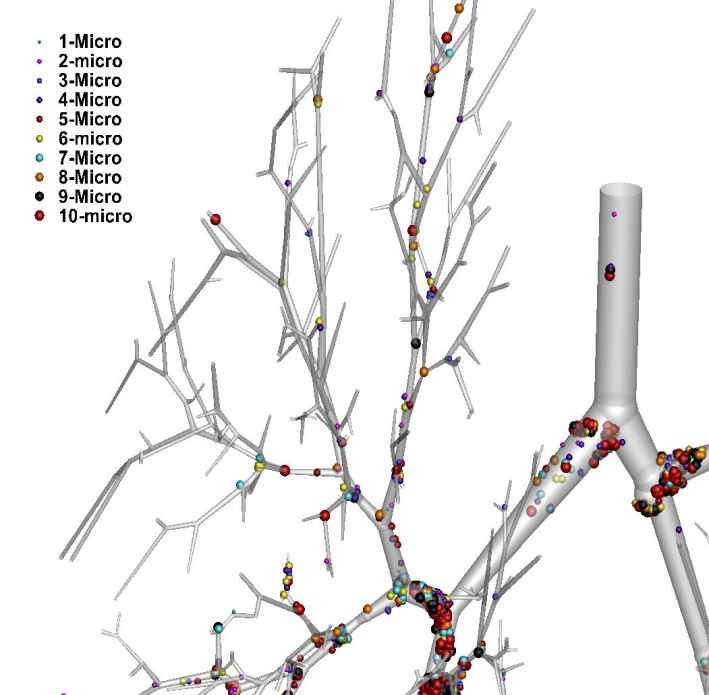 |
| --- | --- |
|  |  |

**Supplementary Figure** 4: Polydisperse particle deposition pattern for 25 lpm flow rate; (a) the overall deposition pattern, (b) deposition at the left upper lobe.

**Supplementary Figure** 5 displays deposition density (deposited particle concentration) comparison of the right and left lung for different flow rates. The comprehensive deposition density curves for different diameter particles are examined. **Supplementary Figures** 5(a, b, c) show the polydisperse particle deposition hot spot for different flow rates. Two different deposition hot spots (G2-G3 and G5-G6) are observed in the right lung at the 9 lpm flow rate. The detail generation definition for the present model can be found in the appendix section. For the 25 lpm flow rate however, a single deposition hot spot is observed in the right lung and the left lung. **Supplementary Figure** 5(c) shows the deposition density comparison for different diameter particles during high flow rate (60 lpm), and a different deposition hot spot is observed for various diameter particles. For larger diameter particle (d_p_ >9μm), G4-G6 is the deposition hot spot in the right lung, whereas G4-G5 is the deposition hot spot in the left lung for all diameter particle. The density curve shows that particle deposition concentration is higher in the upper airways of the right and left lung. The deposition density curve clearly shows that larger diameter particle (d_p_ >7μm) deposition concentration is significantly higher at the upper airways than smaller diameter particles. Greater inertia of microparticles, a highly asymmetric bifurcating airway and the higher flow rate influence the deposition pattern.

| 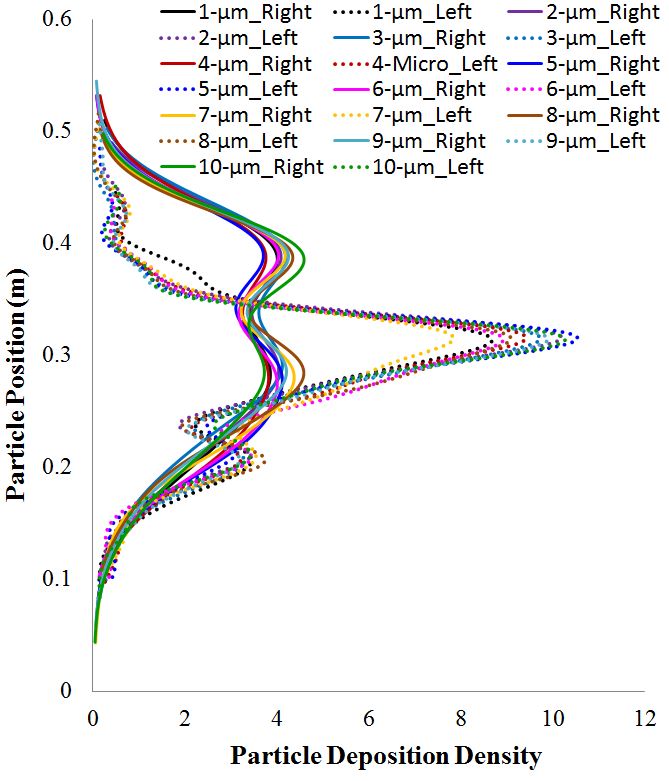  (a) | 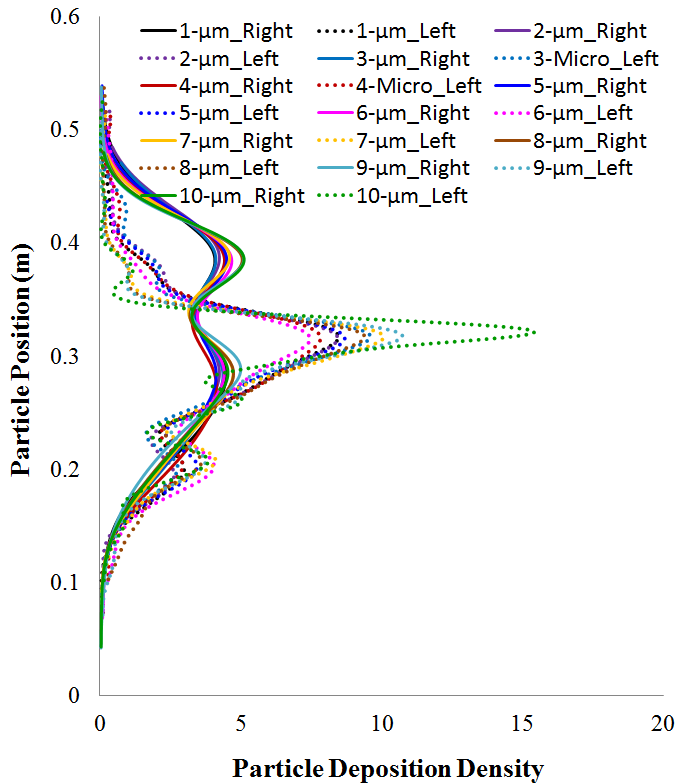  (b) |
| --- | --- |
| 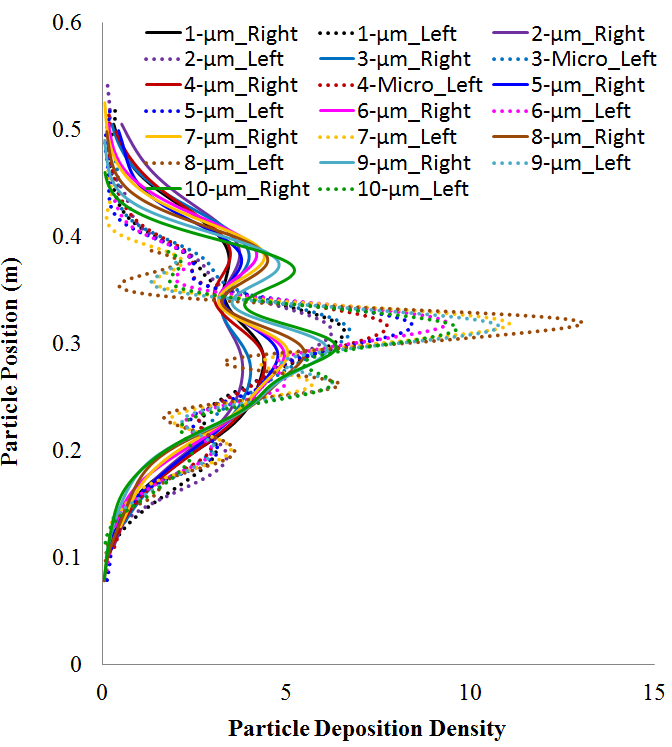  (c) | |

**Supplementary Figure** 5: Particle deposition density comparison at left and right lung for different diameter particles with (a) 9 lpm flow rate, (b) 25 lpm flow rate, (c) 60 lpm flow rate.

| 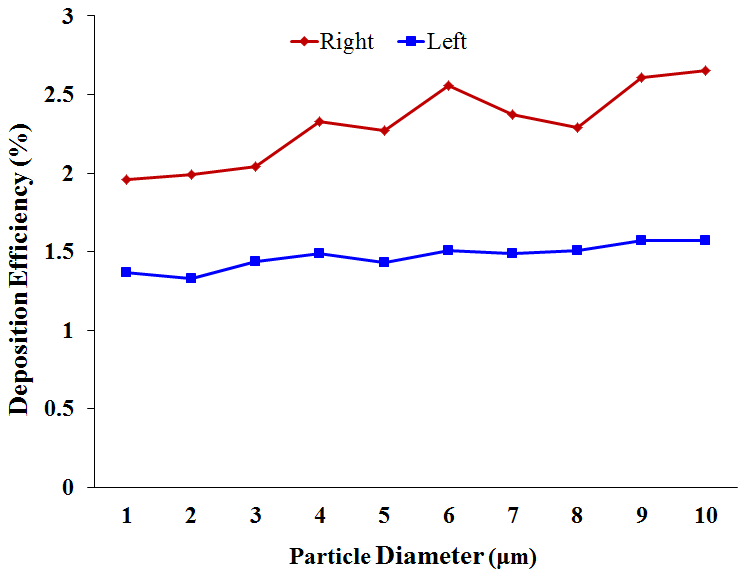  (a) | 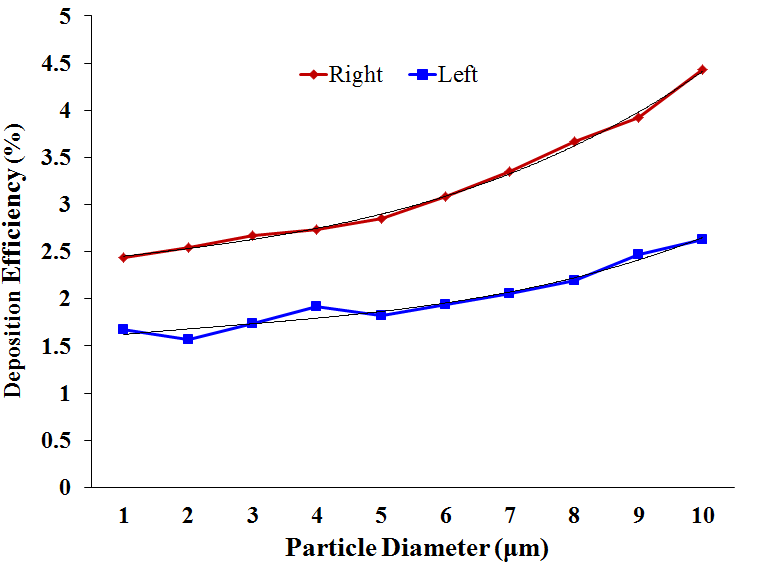  (b) |
| --- | --- |
| 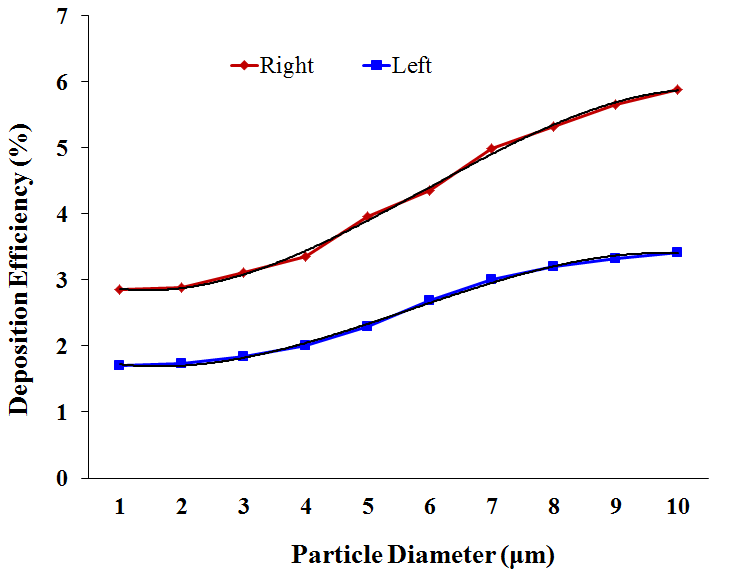  (c) | |

**Supplementary Figure** 6: Polydisperse particle DE comparison in right and left lung for different flow rates; (a) 9 lpm, (b) 25 lpm, and (c) 60 lpm.

Polydisperse particle DE comparison in the right lung and left lung (**Supplementary Figure** 6) for different flow rates is also investigated; however, the overall deposition scenario shows higher DE in the right lung than in the left, regardless of flow rate and particle size. The anatomical asymmetricity of the current 17-generation model and microparticle inertia are the main causes of higher deposition in the right lung. The total flow rate distribution in the right lung is 1.32 times higher than in the left lung at the 9 lpm flow rate. This phenomenon explains the higher deposition in the right lung than in the left lung. At 60 lpm flow rate, the total flow rate distribution in the right lung is 1.3 times higher than in the left lung. Third order analytical polynomial equations are derived from the DE in the right and left lung for 25 lpm and 60 lpm flow rates. The trend line fits well with the DE curve in the left and right lung. The third order polynomial equation for DE in the right and left lung for a 25 lpm flow rate is:

$y_{\text{Right}}=0.0014x^{3}-0.001x^{2}+0.0743x+2.3773$ (16)

$y_{\text{Left}}=0.0012x^{3}-0.0086x^{2}+0.0752x+1.5543$ (17)

The third order polynomial equation for DE in the right and left lung for a 60 lpm flow rate is:

$y_{\text{Right}}=-0.0086x^{3}+0.1521x^{2}-0.389x+3.1143$ (18)

$y_{\text{Left}}=-0.0065x^{3}+0.1105x^{2}-0.3087x+1.929$ (19)

The analytical equations for DE in the right lung and the left lung for different flow rates can be used to predict the polydisperse particle deposition pattern for a whole lung model, as it is nearly impossible to conduct any CFD or experimental study for a whole lung model.

| 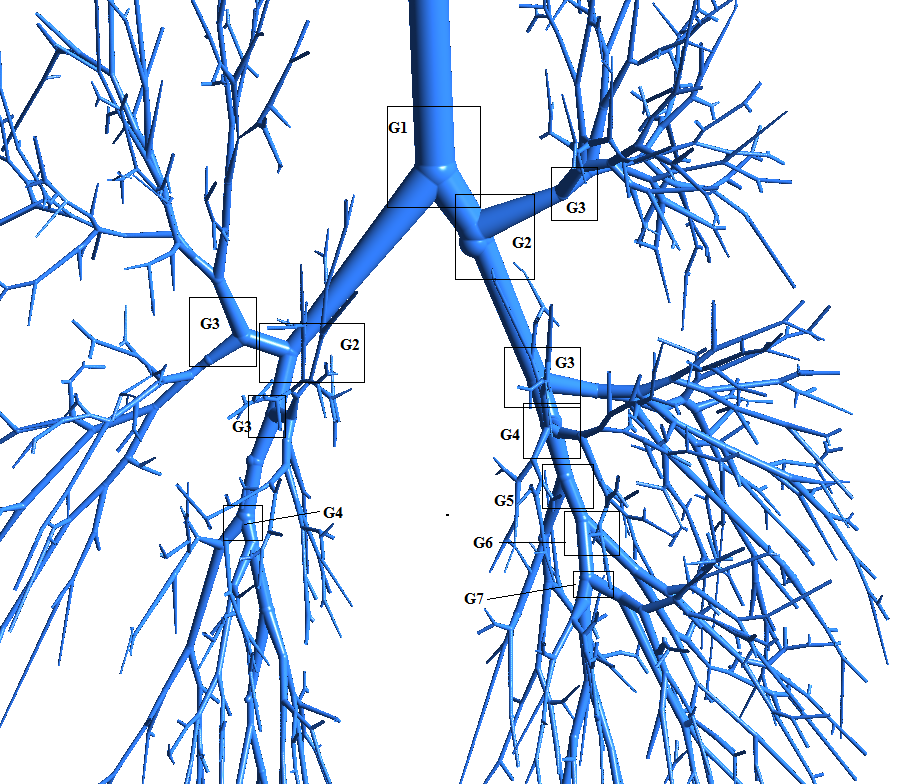 |
| --- |

**Supplementary Figure** 7: Overall generation definition for the present anatomical model.

**Limitation of the Study**

The anatomical model considered possible entire branching pattern (1453 bronchi) and does not consider the extra-thoracic airways. This study assumed zero pressure at the 17-generation outlets. In the real case, there is a small pressure variation at the outlet of the terminal airways for a whole lung model. The current study considered the first 17 generations of the pulmonary airways from the trachea and for this reason; open outlet condition (zero pressure) is used at the outlet. This study does not consider the exhalation effects for particle transport and deposition.
